# Banana Lectin: A Brief Review

**DOI:** 10.3390/molecules191118817

**Published:** 2014-11-17

**Authors:** Senjam Sunil Singh, Sanjenbam Kunjeshwori Devi, Tzi Bun Ng

**Affiliations:** 1 Laboratory of Protein Biochemistry, Biochemistry Department, Manipur University, Canchipur, Imphal 795003, India; E-Mail: sunil_senjam@rediffmail.com; 2 School of Biomedical Sciences, Faculty of Medicine, The Chinese University of Hong Kong, Shatin, Hong Kong, China

**Keywords:** Musa, banana lectins, reverse transcriptase, cancer cell proliferation, macrophage

## Abstract

Lectins are a group of proteins of non-immune origin that recognize and bind to carbohydrates without modifying them. Banana is the common name for both herbaceous plants of the genus *Musa* and for the fruit they produce. They are indeed a promising source for many medicinal applications. Banana lectins have the potential for inhibiting HIV-1 reverse transcriptase activity, suppressing cancer cell proliferation and stimulating macrophage activities. Nevertheless, compared to other plant lectins, there is relatively little information in the literature on banana lectins, particularly with respect to their structure and biological functions. Herein we focus our review on the structure, functions and exploitable properties of banana lectins.

## 1. Introduction

Banana is a major tropical fruit crop distributed in more than 120 countries with an annual production of 102 million tonnes. Banana belongs to the family Musaceae under the order of Zingiberales with two genera, *Musa* L. and *Ensete* Bruce [1]. It is highly diversified throughout the world but has been reported to originate from Southeast Asia [2]. The genus *Musa* consists of around 50 species while *Ensete* has nine species [3]. On the basis of phenotypic traits and basic chromosome number, *Musa* has been divided into four sections namely, Eumusa, Rhodochlamys, Austra-limusa and Callimusa. Most edible bananas that have largely evolved from two wild species, *M. acuminata* Colla and *M. balbisiana* Colla belong to the section Eumusa [4,5].

Traditionally banana has been used for numerous medicinal purposes. For example, banana can be useful in many forms for the treatment of diarrhea, and gastric ulcers, *etc.* Banana is an excellent source of potassium and a number of vitamins [6]. Jain *et al.* [7] investigated the antibacterial and antioxidant activities of different parts of seeded banana fruits *in vitro*. Dried peels, pulps and seeds of the fruit were extracted with hexane, ethyl acetate and ethanol. Antibacterial property of the extracts was evaluated against Gram-positive and Gram-negative bacteria using the disc diffusion technique. The antioxidant activities were determined by the 2,2-diphenyl-1-picrylhydrazyl (DPPH) scavenging system, ferric reducing ability in plasma (FRAP) assays and total phenolic content (TPC) assays [8]. It was shown that the extracts of local seedy banana fruits possessed significant antibacterial and antioxidant activities. Sampath *et al.* [6] also reported that all parts of the banana plant have medicinal applications and at the same time banana lectins exhibit the potential of inhibiting HIV-1 reverse transcriptase activity, suppressing cancer cell proliferation and stimulating macrophage activities. Nevertheless, compared to other plant lectins, very few banana lectins have been structurally characterized or produced in a recombinant form. Therefore the aim of this article is to provide an overview of the structure, functions and exploitable properties of banana lectins.

## 2. BanLec

Lectins are a group of proteins of non-immune origin that recognize and bind to carbohydrates without modifying them [9]. Lectins are known to be important for many biological processes, due to their ability to recognize cell-surface carbohydrates with high specificity. Plant lectins have been model systems for studying protein-carbohydrate recognition, because individually they exhibit high sensitivity and as a group large diversity in recognizing carbohydrate structures. Some of the well-characterized roles of lectins are in cell-cell communication, cancer metastasis, embryogenesis, host-pathogen interactions, and tissue development [10]. A large-scale data acquisition and extensive analysis of sequences and structures of beta-prism-I or jacalin-related lectins (JRLs) have shown that hypervariability in the binding site loops generates carbohydrate recognition diversity [11].

During an investigation of the human antibody response to various foods, Koshte *et al.* [12] observed marked binding of IgG4 to banana (*Musa paradisiaca*) extract from its fruit. In view of the mucilaginous nature of the banana extract, they considered the possibility of non-specific binding to IgG4 and decided to purify the active principle. They found that this binding activity was largely removed by gel filtration on a Sephadex G-75 column and was recovered by elution with sugar (mannose). This clearly indicated the presence of a lectin. They named it as BanLec-I [13].

That was how the banana lectin (BanLec or BL) was first isolated from *Musa paradisiaca* by Koshte and colleagues [12]. They also reported that BanLec is a homodimeric protein that binds mannose and mannose-containing oligosaccharides and functions as a potent T-cell mitogen [9,13]. Later on, Peumans and colleagues cloned and sequenced BanLec (*Musa acuminata*) and confirmed that it is composed of two identical 15-kDa subunits demonstrating specificity toward mannose. Based on sequence homology and secondary structure predictions, they classified BanLec as a member of JRL [14].

Peumans *et al.* [14] identified one of the predominant proteins in the pulp of ripe bananas (*Musa acuminata* L.) and plantains (*Musa* spp.) as a lectin. The identification of these lectins in banana plants and plantains demonstrates that JRLs also occur in monocot species. This point clearly suggests that lectins are actually more widespread among higher plants than is believed.

## 3. Isolation

The banana and plantain lectins are the first documented examples of JRLs, which are present in abundance in the pulp and roots of mature fruits, but are apparently absent from other tissues like peel, leaf and corm. However, Clendennen *et al.* [15] demonstrated that after treatment of intact banana plants with methyl jasmonate, BanLec can be induced in leaves. This clearly indicates that lectin might be expressed as a response to abiotic or biotic stress factors.

The banana and plantain agglutinins (called BanLec and PlanLec, respectively) have been purified in sizeable quantities using a novel isolation procedure, such as by preventing adsorption of the lectins onto insoluble endogenous polysaccharides. Original isolation procedures for BanLec adopted traditional affinity chromatography and acetic acid extraction of overripe bananas, presumably because much of the starch has been degraded and the low pH further reduced starch–lectin interaction. However, Wearne *et al.* [16], reported the added disadvantages of this procedure which include a low yield (10 mg/kg) of purified lectin and it may be due to low solubility of the lectin in an acidic medium, and/or to degradation of lectin during the ripening process.

At the other extreme, one-step purification of lectins using sugar-immobilized gold nanoparticles (SGNPs) was developed by Nakamura *et al.* [17]. With the use of such glycan-coated nanoparticles, a nearly 100-fold greater yield (1 µg/mg) was achieved. They used the 60% ammonium sulfate fraction obtained from the crude plant extract, incubated it with SGNPs overnight at 4 °C to allow to form aggregate of lectin and SGNPs. The lectin was finally dissolved by adding inhibitory sugars. These sugars are similar or identical to the non-reducing sugar moieties on the SGNPs. This procedure reflects the ability of the nanoparticles, coated with a high density of reactive mannosyl/glucosyl groups, to successfully compete with the starch of banana pulp for binding with the lectin [16,17].

## 4. Structures

Plant lectins have been classified into five structural families [18] in terms of their polypeptide folds. Of these, the β-prism I fold in lectins was first characterized by Sankaranarayanan *et al.* in jacalin from jackfruit seeds [19]. The other lectin from jackfruit seeds with different sugar specificity, artocarpin, also exhibits the same fold [18]. Originally, the β-prism I fold was thought to be characteristic of Moraceae lectins. However, lectins from other families were subsequently shown using X-ray analysis to display the same fold: e.g., calsepa from *Calystegia sepium* (family Convolvulacea) and heltuba from *Helianthus tuberosus* (family Asteraceae) [18]. On the basis of sequence similarity, Peumans *et al.*, have also disclosed that banana lectin also manifests the same β-prism I fold [14].

Peumans *et al.* [14], showed that both BanLec (banana lectin) and PlanLec (plantain lectin) are dimeric proteins composed of two identical 15-kDa subunits each of which contains 141 amino acid residues. By molecular cloning and molecular modelling studies of the protein they also revealed that BanLec has sequence similarity to previously described lectins of the family of JRLs, possessing the same overall fold and three-dimensional structure [9]. Raval *et al.* [11], classified the JRLs family into three categories based on their quaternary structures. The first group contains jacalin and artocarpin, the second group contains calsepa and the third group contains heltuba [19]. Meagher *et al.* [9], proposed the fourth structural category of JRLs which contains the BanLec because its quaternary structure shows a unique dimer [9]. Dimeric banana lectin and calsepa, tetrameric artocarpin and octameric heltuba are mannose-specific β-prism I fold lectins with nearly the same tertiary structure [20].

Khan *et al.* [21], proposed the ribbon-shaped structure in BanLec (homodimeric protein), in which each subunit has a single Trp residue at position 10. Also each subunit has twelve β strands arranged in a β-prism-I fold. More importantly, the structure of BanLec obtained by X-ray crystallography revealed the presence of a second sugar binding site. The residues involved in the second site are common to other lectins in this family, potentially signaling a new group of mannose-specific jacalin-related lectins (mJRL) with two sugar binding sites *i.e.*, two primary sugars-binding sites (for the same type of sugar residue) per subunit of banana lectin. Molecular cloning revealed that BanLec has sequence similarity to previously described lectins of the family of JRL, and according to molecular modeling studies it has the same overall fold and three-dimensional structure [14,22].

BanLec is reported to be the only one among the lectins of known structure with β-prism I fold to exhibit more than one primary combining site per subunit [22]. It is known that all forms of monocot plants invariably have the β-prism II fold lectins with three carbohydrate-binding sites in each subunit/domain. Whilst β-prism I fold lectins of known structure are all from dicots and they exhibit one carbohydrate-binding site per subunit/domain. However the BanLec which is from a monocot, has β-prism fold I lectin structure with two very similar carbohydrate-binding sites [23].

BanLec furnishes a unique example in proteins where subunit association is not a result of primary hydrophobic interactions. It was reported by Gupta *et al.* [22], that BanLec is very stable and is denatured only at high concentrations of chaotropic agents. The main reason behind the stability of banana lectin dimer might be attributed to strong hydrogen bonds at the dimeric interface along with the presence of water bridges.

## 5. BanLec—Properties and Binding Specificity

BanLec, the mannose-specific lectin from banana (*Musa acuminata*, or polyploidy hybrid cultivars thereof), recognizes terminal α-d-mannosyl/glycosyl units, internal α-1,3-mannosyl/glucosyl units, and reducing terminal β-1,3-glucosyl units such as those occuring in laminarin, the polysaccharide from the brown alga *Laminaria digitata.* This specificity renders it of potential value in medicinal applications, such as inhibition of HIV replication through interaction with its gp120 coat glycoprotein, as has recently been demonstrated [16,24].

The crystallographic, modeling, and simulation studies conducted by Sharma *et al.* [25], provide fresh insights into the geometrical details of banana lectin–carbohydrate interactions. The lectin associates at the primary binding site with disaccharides involving mannose or glucose residues on α-1,3 linkage towards the non-reducing end. The reducing end occupies the site when the linkage is β-1,3. Both arrangements appear to be allowed to nearly the same extent when the linkage is α-1,2. Interestingly α-1,3-linked disaccharides cannot bind to galactose-specific β-prism I fold lectins, with the non-reducing end at the primary site on account of steric clashes with an aromatic residue that is present only when the lectin is galactose-specific. BanLec is unique in its specificity for internal β-1,3 linkages as well as β-1,3 linkages at the reducing termini [26]. BanLec also explicitly binds to terminal nonreducing α-d-glucosyl/mannosyl units of oligo-/polysaccharide chain ends [21,27,28]. Mo *et al.* [27], performed extensive experiments in order to find out the exact specificity of BanLec (*Musa acuminata*) and lectin from the closely related plantain (*Musa* spp.) by using the techniques of quantitative precipitation, hapten inhibition of precipitation, and isothermal titration calorimetry and showed that they are mannose/glucose binding proteins with a preference for the α-anomeric form of these sugars. Both generate precipitin curves with branched chain α-mannans (yeast mannans) and α-glucans (glycogens, dextrans, and starches), but not with linear α-glucans containing only α-1,4- and α-1,6- glucosidic bonds (isolichenan and pullulan). Another novel observation was also made that banana and plantain lectins recognize their specific binding sites that occur in the linear polysaccharides of elsinan and nigeran, which is unlike that of other mannose/glucose binding lectins (concanavalin A and lectins from pea and lentil).

Again Goldstein *et al.* [29], further showed that this lectin also binds to the reducing glucosyl groups of β-1,3-linked glucosyl oligosaccharides (e.g., laminaribiose oligomers). Additionally, banana lectin also recognizes β-1,6-linked glucosyl end groups (gentiobiosyl groups) which occur in many fungal β-1,3/1,6-linked polysaccharides. This behavior clearly distinguishes the banana lectin from other mannose/glucose binding lectins, such as concanavalin A and the pea, lentil and *Calystegia sepium* lectins. Therefore BanLec is unique in its recognition of internal glucosyl/mannosyl residues linked α-1,3-, but not β-1,3-, and especially in its recognition of the reducing sugar unit of laminaribiose (Glcβ1,3Glc) and its higher homologues [27,28,29].

An X-ray analysis of BanLec was undertaken by Singh *et al.* [18,30], owing to its interesting sugar-binding properties and its similarity to and differences from other JRLs. It was subsequently shown that BanLec is mannose/glucose-specific, with a preference for α-anomeric forms. In addition to α-1-3-linked glucosyl residues, BanLec recognizes reducing glucosyl groups of β-1-3-linked glucosyl oligosaccharides and β-1-6-linked glucosyl end-groups [18,27,29]. Cheung *et al.* [31], reported another banana lectin from Del Monte bananas (*Musa acuminata*) having fructose as the most potent inhibitory sugar.

## 6. Biological Properties

BanLec is a mannose-specific lectin which readily agglutinates rabbit erythrocytes. BanLec exhibits a strong mitogenic activity towards murine T-cells. The relevance of the mitogenicity of the banana lectin in terms of their physiological role and the impact on food safety was also discussed by Peuman *et al.* [14], Singh *et al.* [18] and Sankaranarayanan* et al.* [19]. BanLec is also reported to act as a potent inhibitor of HIV replication [21,32]. This lectin binds to high mannose carbohydrate structures, including those found on viruses containing glycosylated envelope proteins such as human immunodeficiency virus type-1 (HIV-1). Therefore, Swanson *et al.* [32], hypothesized that BanLec might inhibit HIV-1 through binding of the glycosylated HIV-1 envelope protein, gp120. They also found that BanLec inhibits primary and laboratory-adapted HIV-1 isolates of different tropisms and subtypes with IC_50_ values in the low nanomolar to picomolar range. The mechanism of BanLec-mediated antiviral activity was explained by their data indicating that BanLec inhibits HIV-1 infection by binding to the glycosylated viral envelope and blocking cellular entry. They also compared the relative favorable anti-HIV activity of BanLec towards other anti-HIV lectins, such as snowdrop lectin and griffithsin, and to anti-HIV drugs currently in clinical use (T-20 and maraviroc). Based on these results, they concluded that BanLec is a potential anti-viral microbicide that could be used to prevent sexual transmission of HIV-1.

BanLec is analogous to receptors present in the plasma and blood cells of crayfish and insect species which recognize and bind to β1,3-glucans [9]. A mannose-specific plant lectins from the Amaryllidaceae family also showed microbicidal property for preventing HIV infection [33]. *Balzarini et al.* [24], showed that the α-(1-3)-d-mannose- and α-(1-6)-d-mannose-specific agglutinins (lectins) from *Galanthus nivalis*, *Hippeastrum hybrid, Narcissus pseudonarcissus*, and *Listera ovata* inhibited infection of MT-4 cells by human immunodeficiency virus types 1 and 2 (HIV-1 and HIV-2) and simian immunodeficiency virus. They also suggested that these plant lectins interfere with an event in the HIV replicative cycle (fusion process). It explains why BanLec possesses anti-HIV property as it also has specificity towards similar internal α-1-3-linked mannosyl/glucosyl residues.

Cheung *et al.* [31], reported a lectin from Del Monte banana (*Musa acuminata*) with cytokine-inducing activity in murine splenocytes. It was able to induce the expression of cytokines such as interferon-gamma, tumor necrosis factor-alpha, and interleukin-2. The lectin also inhibited proliferation of leukemia (L1210) cells and hepatoma (HepG2) cells and the activity of HIV-1 reverse transcriptase. Wong *et al.* [34], also isolated another banana lectin from *Musa basjoo* cv. (Emperor Banana) demonstrating stimulatory effects on uptake of [3H-methyl]-thymidine by mouse splenocytes and nitric oxide production by mouse macrophages. The lectin inhibited proliferation of leukemia cells (L1210) and the activity of HIV-1 reverse transcriptase. Hinge *et al.* [35], and Bajaj *et al.* [36], showed that with respect to gene expression as well as the signaling mechanisms involved, BanLec from *Musa paradisiaca* produced an adipogenic action on mesenchymal cells like to that mediated by insulin. They also suggested that this banana lectin may serve as micronutrients for therapeutic purposes in hematological deficiencies. In another paper, Bajaj *et al.* [36], showed that BanLec interacted with insulin receptors on M210B4 cells and induced a mitogen-activated protein kinase (MEK)-dependent ERK signaling in these cells. Biological properties of various reported BanLec are summarized in Table 1.

**Table 1:** Biological properties of various reported banana lectins.

Name	Source	Specificity	Biological Properties	Authors
BanLec	*Musa acuminata*	Mannose binding	Inhibits HIV-1 infection by binding to the glycosylated viral envelope and blocking cellular entry	Swanson [32]
BanLec	*Musa paradisiaca*	Mannose binding	Stimulates T-cell proliferation	Koshte [12]
BanLec	*Musa paradisiaca*	Mannose binding	Adipogenic effect on mesenchymal cells similar to that mediated by insulin. Induces a mitogen-activated protein kinase (MEK)-dependent ERK signaling in M210B4 cells.	Hinge [35]; Bajaj [36]
BanLec	*Musa basjoo* cv	Glucose/mannose-specific	Macrophage-stimulating, antiproliferative and HIV-1 reverse transcriptase inhibiting activities.	Wong [34]
BanLec	*Musa acuminata*	Mannose/glucose binding protein	T-cell mitogen and induces the formation of IgG4 antibodies.	Mo [27]; Winter [28]
BanLec	*Musa acuminata*	Mannose binding (with two sugar binding sites)	Functions as a potent T-cell mitogen	Meagher [9]
Recombinant BanLec (rBanLec)	Produced in *E. coli*	Similar specificity with native BanLec from *Musa acuminate*	Having potential for modulation of the immune response. Increases T lymphocyte proliferation and secretion of interferon-gamma by mouse splenocytes.	Gavrovic-Jankulovic [26]; Dimitrijevic [37]; Dimitrijevic [38]
BanLec	*Musa acuminata*	Fructose as most potent inhibitory sugar	Mitogenic in murine splenocytes and inducing cytokines interferon-gamma, tumor necrosis factor-alpha, and interleukin-2 expression in splenocytes. Inhibits proliferation of leukemia (L1210) and hepatoma (HepG2) cells and the activity of HIV-1 reverse transcriptase.	Cheung [31]

## 7. Recombinant BanLec

Gavrovic-Jankulovic *et al.* [26], cloned the gene of banana lectin, and a recombinant protein was produced in *Escherichia coli*. The cDNA obtained revealed a novel banana lectin isoform, with an open reading frame of 426 nucleotides, encoding a cytoplasmatic protein of 141 amino acids (Table 2). The molecular mass and sugar specificity of the recombinant lectin was comparable with the natural banana lectin. However, Wearne *et al.* [16], observed that recombinant BanLec (rBanLec) expressed in *Escherichia coli*, although chemically and functionally identical to native BanLec, differed slightly in its apparent molecular size, probably because rBanLec may be folded differently from the native protein. This rBanLec is a useful reagent for glycoproteomics and lectin microarrays, with a potential for modulation of the immune response (a potent modulator of the proliferation response in CD3+, CD4+, and CD8+ populations of human PBMCs) [16].

Dimitrijevic *et al.* [37], conducted studies on thermal stability and digestibility of rBanLec on simulated gastro-intestinal fluid as well as simulated intestinal fluid and concluded that rBanLec is a good candidate for the novel bioadhesive lectin-based drug delivery systems to the gastro-intestinal tract (GIT). Dimitrijevic *et al.* [38], also used rBanLec to study its effects as a mucosal immunostimulator * in vivo* in mice and found that it is stable in the mouse digestive tract, where it specifically interacts with the mucosal surfaces which can be inhibited by the addition of glucose. They also suggested that rBanLec is a potential oral adjuvant to the gastrointestinal tract.

**Table 2:** Structural aspects of various reported banana lectins.

Name	Source	Structure	Amino Acid Sequence	Authors
BanLec	*Musa paradisiac*	Homodimeric, ~13-kDa subunits	_	Koshte [12]
BanLec	*Musa acuminata* L.	Homodimeric, 15-kDa subunits	141aa residues	Peumans [14]
BanLec	*Musa basjoo* cv	Homodimeric, 15-kDa subunits	*N*-terminal amino acid sequence similar to lectins from other *Musa* species	Wong [34]
rBanLec	Produced in *E. coli*	cDNA—426 nucleotides 15.9-kDa	141aa residues with *N*-terminal sequences of TGAIKVGA	Gavrovic-Jankulovic [26]; Dimitrijevic [37]

## 8. Discussion

BanLec are homodimeric and display a molecular weight of about 13–15-kDa (Table 2). Most of them are mannose-specific [9,27,28] except for one species, *Musa acuminata*, which has fructose [31] as the most potent inhibitory sugar. All of them belong to one family of lectins that is JRLs. BanLec differs from other related members of the JRLs family in term of quaternary structure that involves a unique dimer and is included in the fourth category of JRLs family. Banana lectins are found to possess many exploitable biological properties like HIV-1 reverse transcriptase inhibiting activity, antiproliferative activity toward cancer cells and macrophage stimulating activity (Table 1).

It is well known that bananas are rich in potassium and beneficial due to their health-promoting effects [31]. Because the binding, toxicity, and anti-HIV activity of lectins vary, the identification of novel anti-viral lectins, such as BanLec, will further increase the possibility of successful development of a lectin-based anti-HIV microbicide [32]. rBanLec is also a candidate for the novel oral adjuvant to the gastrointestinal tract. Hence, potential applications could also be explored for rBanLec in view of its safety for human and animal consumption, its resistance to proteolytic degradation and binding to mucosal surfaces, which could increase the availability of therapeutics and enhance or redirect an immune response against target immunogens [26,37,38]. Moreover BanLec is an excellent tool for glycoprotein research [12].

There are various reports on the biomedical applications of BanLec. However, due to the low yield of BanLec from their sources of when conventional methods of isolation are employed, BanLec does not find its glamour in industrial applications. Therefore it is high time to focus more on the development of new methods like genetic engineering for the mass production of rBanLec. Also, more sequence information on BanLec would be helpful for structural studies and functional characterization. BanLec has potential applications based on immunomodulatory, antiproliferative, and antiviral/antimicrobial activities, *etc.* Nevertheless, there is still a need for other techniques like proteomics and genomic analysis to explore various aspects of these important biomolecules.
